# Template‐Directed Copying of RNA by Non‐enzymatic Ligation†

**DOI:** 10.1002/anie.202004934

**Published:** 2020-08-13

**Authors:** Lijun Zhou, Derek K. O'Flaherty, Jack W. Szostak

**Affiliations:** ^1^ Howard Hughes Medical Institute Department of Molecular Biology Center for Computational and Integrative Biology Massachusetts General Hospital Boston MA 02114 (USA), E; ^2^ Present address: Alnylam Pharmaceuticals Cambridge MA 02142 USA

**Keywords:** non-enzymatic ligation, origins of life, prebiotic chemistry, ribonucleotides, RNA replication

## Abstract

The non‐enzymatic replication of the primordial genetic material is thought to have enabled the evolution of early forms of RNA‐based life. However, the replication of oligonucleotides long enough to encode catalytic functions is problematic due to the low efficiency of template copying with mononucleotides. We show that template‐directed ligation can assemble long RNAs from shorter oligonucleotides, which would be easier to replicate. The rate of ligation can be greatly enhanced by employing a 3′‐amino group at the 3′‐end of each oligonucleotide, in combination with an N‐alkyl imidazole organocatalyst. These modifications enable the copying of RNA templates by the multistep ligation of tetranucleotide building blocks, as well as the assembly of long oligonucleotides using short splint oligonucleotides. We also demonstrate the formation of long oligonucleotides inside model prebiotic vesicles, which suggests a potential route to the assembly of artificial cells capable of evolution.

## Graphical abstract

**Non‐enzymatic RNA Copying**: Enhanced non‐enzymatic ligation allows the rapid copying of long RNA template and short RNA splinted templates, thus suggesting a potential route to the assembly of artificial systems capable of evolution.

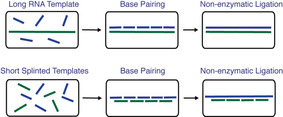

## Introduction

The RNA‐world hypothesis posits an early stage in the evolution of life during which RNA was both the carrier of genetic information and the catalyst of cellular reactions.^1^, ^2^, ^3^, ^4^ This model is supported by the in vitro evolution of ribozymes with RNA polymerase activity.^5^, ^6^, ^7^ However, prior to the emergence of the first RNA replicase ribozyme, genetic polymers must have relied on non‐enzymatic self‐replication processes to survive.^8^ The current lack of an efficient pathway for non‐enzymatic RNA replication is the main obstacle towards building a model protocell capable of growth, division, and therefore Darwinian evolution.^9^ Recent progress in template‐copying chemistry with activated mononucleotide substrates has enabled the copying of mixed‐sequence RNA templates up to 7 nucleotides (nt) long in solution and 5 nt long inside a model protocell.^10^, ^11^ In more artificial scenarios, it has been possible to copy bead‐immobilized templates up to 12 nt in length, thereby completing a functional ribozyme sequence.^12^ In spite of this progress, it is not yet possible to copy, in an effective and prebiotically plausible manner, RNA templates long enough to encode ribozymes that might enable RNA‐catalyzed self‐replication processes.^13^, ^14^

As an alternative to mononucleotide polymerization, templates might be copied by the ligation of short oligomers to generate longer products.^15^ However, the slow rate and low yield of template‐directed RNA ligation^12^, ^16^ has precluded the copying of long functional sequences by ligation. In an effort to overcome this problem, the Orgel and von Kiedrowski laboratories used 1‐ethyl‐3‐(3‐dimethylaminopropyl) carbodiimide (EDC)‐driven ligation of oligonucleotides with 3′‐amino‐RNA or 3′‐phosphoryl‐DNA to demonstrate the copying of tetramer and hexamer templates by the ligation of dimer and trimer substrates, respectively.^17^, ^18^ Unfortunately, no example of long RNA copying by chemical ligation has been reported in the subsequent 30 years. Despite the challenges, template copying by ligation remains attractive because it requires fewer reaction steps to copy a template of a given length. A ligation approach could also overcome other problems that are severe for monomer polymerization, such as copying through secondary structures, copying A/U rich sequences,^19^ and copying the last nucleotide of a template.^20^ Moreover, short oligomers would have been prebiotically accessible, as inevitable products of reactions between activated monomers both on and off template.^21^ In light of these advantages, further studies of non‐enzymatic ligation are needed to evaluate the potential of ligation mediated pathways for template assembly and copying.

Herein, we explore the use of highly reactive oligonucleotide substrates in non‐enzymatic template directed ligation. We used short oligoribonucleotides terminated with 3′‐amino‐2′,3′‐dideoxy‐ribonucleotides as substrates because the 3′‐amino moiety is a stronger nucleophile than the 3′‐hydroxyl of ribonucleotides. Although there is no evidence that 3′‐amino‐2′,3′‐dideoxy‐ribonucleotides were prebiotically available, they are an excellent model for the study of how RNA oligonucleotides might behave if the nucleophilicity of the 3′‐hydroxyl could be enhanced, for example by assistance from small molecules or peptides capable of delivering a catalytic metal ion to the reaction center. 3′‐Amino nucleotides and oligonucleotides have been previously employed for origin of life studies.^22^, ^23^ For instance, we have recently shown that 3′‐amino‐2′,3′‐dideoxy‐ribonucleotides activated with 2‐aminoimidazole polymerize efficiently on RNA templates, both in solution and within vesicular compartments,^24^ enabling the copying of templates up to 25 nt in length. The Krishnamurthy group has reported the generation of homogeneous backbone oligonucleotides from heterogeneous sugar‐backbone templates, using the ligation of 3′‐amino oligonucleotides.^25^ However, no investigation of the kinetics of the ligation reaction has been reported to date. Here we report our kinetic studies of the non‐enzymatic ligation of both all RNA and 3′‐amino terminated oligonucleotides, as well as multistep ligations of 3′‐amino oligonucleotides. This work is complementary to our previous studies of monomer polymerization. While monomers must form an imidazolium‐bridged dinucleotide intermediate prior to the primer extension step,^26^, ^27^, ^28^ ligation proceeds through a direct S_N_2‐type reaction. Studies of both monomer polymerization and oligonucleotide ligation will help us to better understand how a hereditary material could replicate prior to the advent of ribozymes.

## Results and Discussion

**Figure 1:**
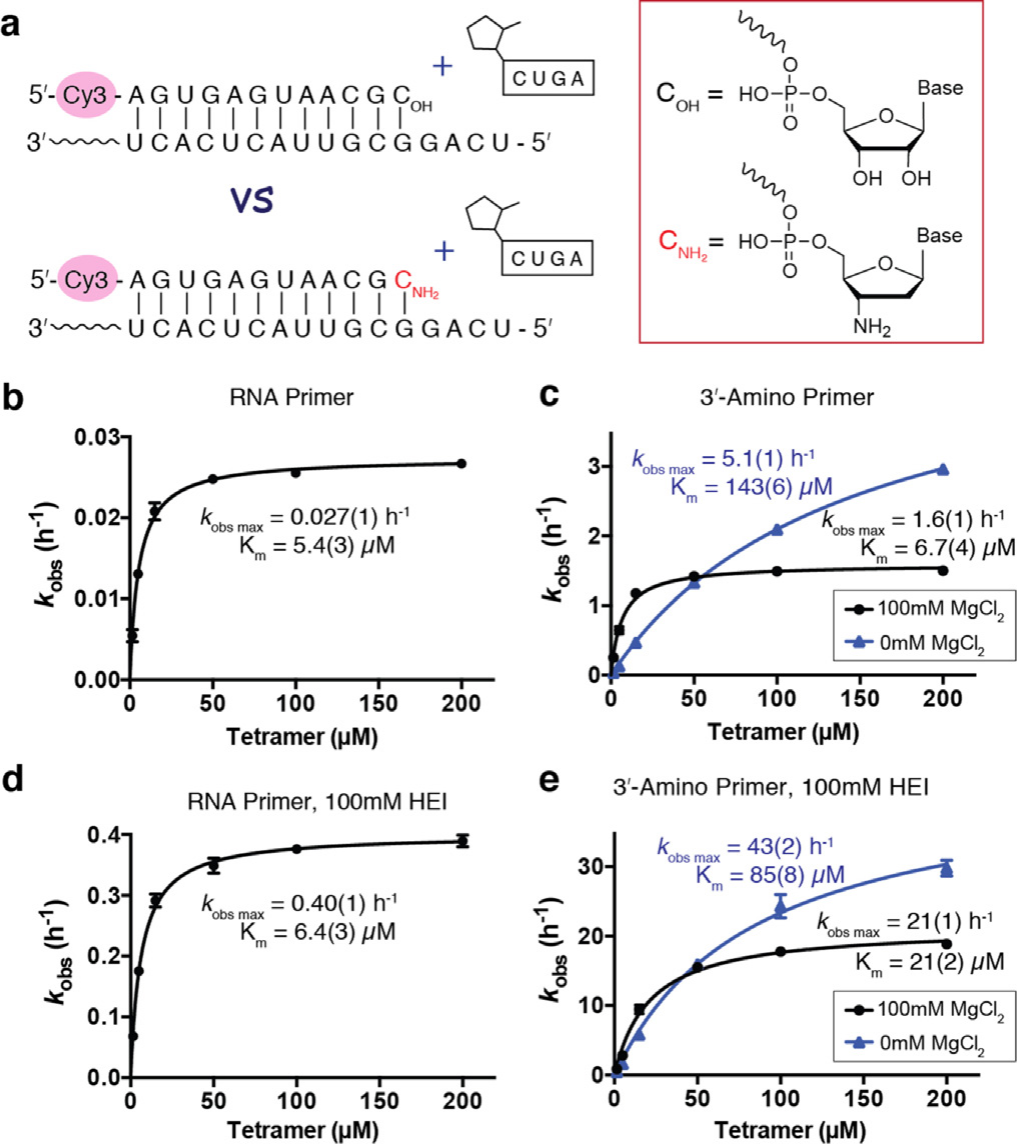
Kinetic studies of non‐enzymatic ligation reactions. a) Illustration of experimental design. The 5′‐fluorescently labelled primer contains either a ribonucleotide (C_OH_) or a 3′‐amino‐2′,3′‐dideoxyribonucleotide (C${}_{\mathrm{NH}{}_{2}}$ ) at the 3′‐terminus. The template is complementary to the primer and has a 5′‐UCAG overhang. The ligator is 2‐MeImp‐CUGA. All experiments were carried out with 2 μm primer, 4 μm template, 200 mm HEPES, pH 8.0, and 100 mm MgCl_2_ unless otherwise noted, and with the indicated concentration of the tetramer ligator. Excess template was used to ensure that all primer was template bound and to avoid non‐templated ligation reactions. b–e) Ligator‐concentration dependence of the ligation rate under different conditions: b) RNA primer; c) 3′‐amino primer; d) RNA primer with 100 mm HEI; e) 3′‐amino primer with 100 mm HEI. Black circles=100 mm MgCl_2_. blue triangles=no MgCl_2_. Data points are reported as the mean±s.d., *n*≥3.

To better understand the effects of a 3′‐amino versus a 3′‐hydroxyl nucleophile on the rate of template‐directed oligonucleotide ligation, we began by quantitatively assessing the kinetics of these ligation reactions. Using an RNA primer containing either a canonical ribonucleotide or a 3′‐amino‐2′,3′‐dideoxy‐ribonucleotide residue at the 3′‐end (Figure 1 a and Figure S1 in the Supporting Information), we measured the rate of primer ligation as a function of concentration of the ligator, in this case a 2‐methylimidazole activated RNA tetramer. The observed maximal rate (*k*_obs max_) of the RNA ligation reaction in the presence of 100 mm Mg^2+^ was 0.027 h^−1^ (Figure 1 b, Figure S2a). Under the same 100 mm Mg^2+^ conditions, substitution of the last nucleotide on the RNA primer with a 3′‐amino‐2′,3′‐dideoxy‐ribonucleotide enhanced the ligation rate by over 50‐fold; giving a *k*_obs max_ of 1.6 h^−1^ (Figure 1 c, Figure S2b). Notably, without Mg^2+^, *k*_obs max_ reached an even higher value of 5.1 h^−1^ (Figure 1 c, Figure S2c). Our results show that Mg^2+^ accelerates the N3′‐P5′ ligation rate only at concentrations of the ligator lower than 50 μm. When the concentration of the ligator is sub‐saturating, Mg^2+^ presumably stabilizes duplex formation and improves the *k*_obs_. At higher, saturating concentrations of tetramer, Mg^2+^ causes the ligation rate to decline for unknown reasons.

Notably, 2‐aminoimidazole is a superior leaving group in non‐enzymatic RNA polymerization due to its ability to stabilize the critical imidazolium‐bridged intermediate dinucleotide.^10^, ^27^ However, 2‐aminoimidazole is intrinsically a worse leaving group compared to 2‐methylimidazole, as illustrated by the slower rate of hydrolysis of 2‐aminoimidazole activated monomers.^27^ In contrast to its benefit in mononucleotide polymerization, 2‐aminoimidazole is not as good a leaving group as 2‐methylimidazole in the case of N3′‐P5′ ligation (Figure S3).

The organocatalyst 1‐(2‐hydroxyethyl)imidazole (HEI) is known to enhance the rate of non‐enzymatic polymerization of imidazole activated monomers.^29^, ^30^ HEI is thought to act as a nucleophilic catalyst that exchanges with the 2‐methylimidazole moiety on the activated nucleotides to generate a more reactive imidazolium leaving group. As expected, the presence of HEI increased the rate of RNA ligation in the presence of 100 mm Mg^2+^ by more than ten‐fold, such that *k*_obs max_ reached 0.40 h^−1^ (Figure 1 d, Figure S4a). Similarly, HEI also increased the rate of the N3′‐P5′ ligation by more than ten‐fold to a *k*_obs max_ of 21 h^−1^ in the presence of 100 mm Mg^2+^, and 43 h^−1^ in the absence of Mg^2+^ (Figure 1 e, Figure S4b,c).

**Figure 2:**
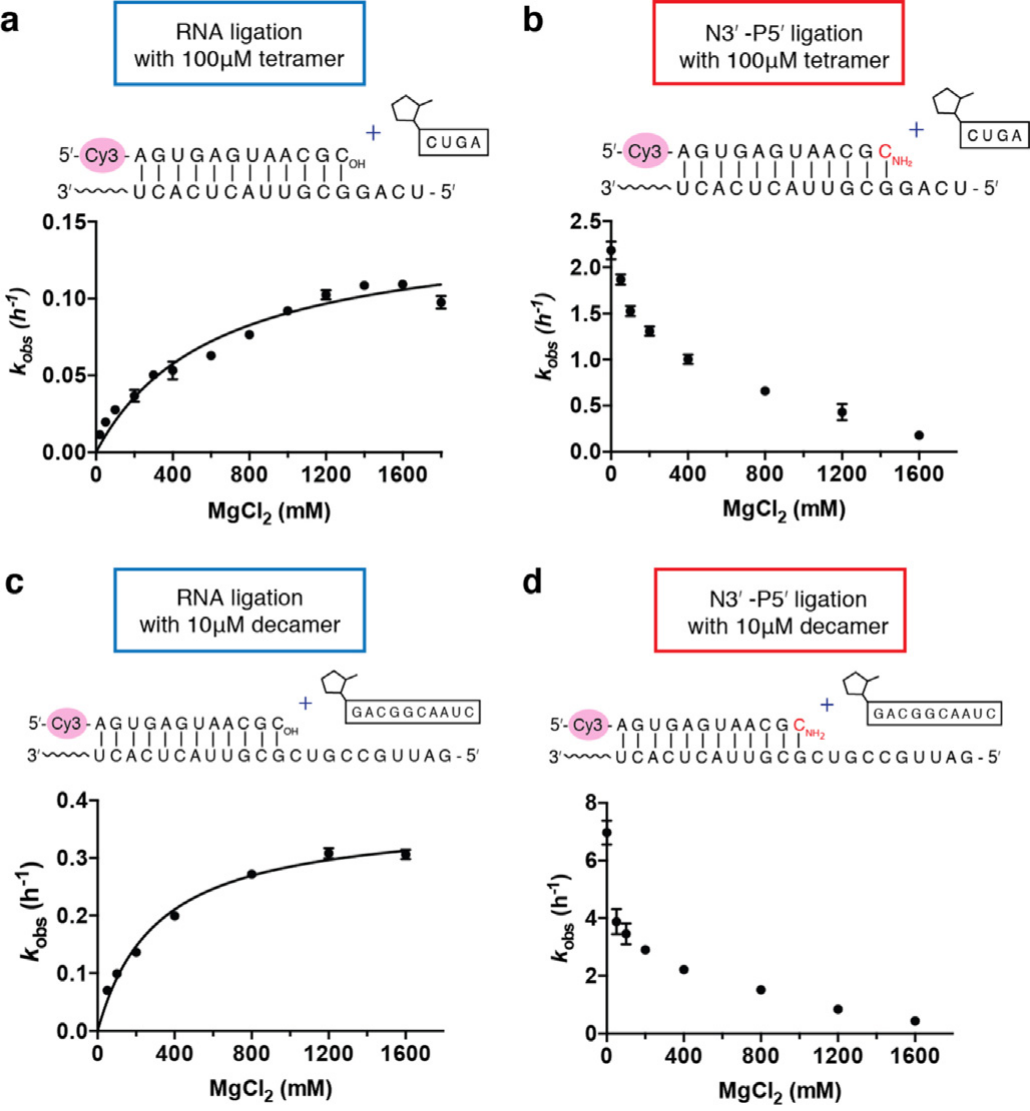
Rates of oligonucleotide ligation reactions as a function of Mg^2+^ concentration. a) Ligation rates of RNA primer with 100 μm tetramer (2‐MeImp‐CUGA). b) Ligation rates of 3′‐amino terminated primer with 100 μm tetramer (2‐MeImp‐CUGA). c) Ligation rates of RNA primer with 10 μm decamer (2‐MeImp‐GACGGCAAUC). d) Ligation rates of 3′‐amino terminated primer with 10 μm decamer (2‐MeImp‐GACGGCAAUC). All experiments were carried out with 2 μm primer, 4 μm template, 200 mm HEPES, pH 8.0, 100 μm tetramer or 10 μm decamer, and MgCl_2_ at the indicated concentration. Data are reported as the mean±s.d. from triplicate experiments. Representative PAGE data are shown in Figures S5–S8.

To further examine the effects of Mg^2+^ on RNA ligation, we examined ligation reactions in the presence of different concentrations of Mg^2+^. To minimize the effect of Mg^2+^ on ligator binding, we performed reactions at saturating ligator concentrations, for example, 100 μm tetramer ligator or 10 μm decamer ligator. Mg^2+^ is thought to assist RNA ligation by deprotonating the 3′‐hydroxyl nucleophile.^31^ RNA ligation in the absence of Mg^2+^ was too slow to measure, while the ligation rate continued to increase up to approximately 0.1 h^−1^ at 1.6 m Mg^2+^ for the tetramer ligator, and around 0.3 h^−1^ at 1.2 m Mg^2+^ for the decamer ligator (Figure 2 a,c).

We also examined the effect of varying Mg^2+^ concentration on ligation with the 3′‐amino primer to provide a direct comparison with the all RNA primer. In contrast to our observations with the RNA primer, we find that the *k*_obs_ of N3′‐P5′ ligation decreases as the concentration of Mg^2+^ increases (Figure 2 b and d). The inhibition of N3′‐P5′ ligation by Mg^2+^ cannot be explained by accelerated ligator hydrolysis by Mg^2+^ because the hydrolysis rates of activated tetramer with or without 100 mm Mg^2+^ are two orders of magnitude slower than the rate of N3′‐P5′ ligation (Figure S9). Thus, we conclude that Mg^2+^ is beneficial for N3′‐P5′ ligation only when the concentration of the ligator is too low to afford sufficient binding to the template. Otherwise, the presence of Mg^2+^ decreases the rate of the reaction. Notably, the ligation rates of the decamer ligator are higher than the rates of the tetramer ligator. This effect may reflect a more stable and favorable conformation of decamer/template duplex.

**Figure 3:**
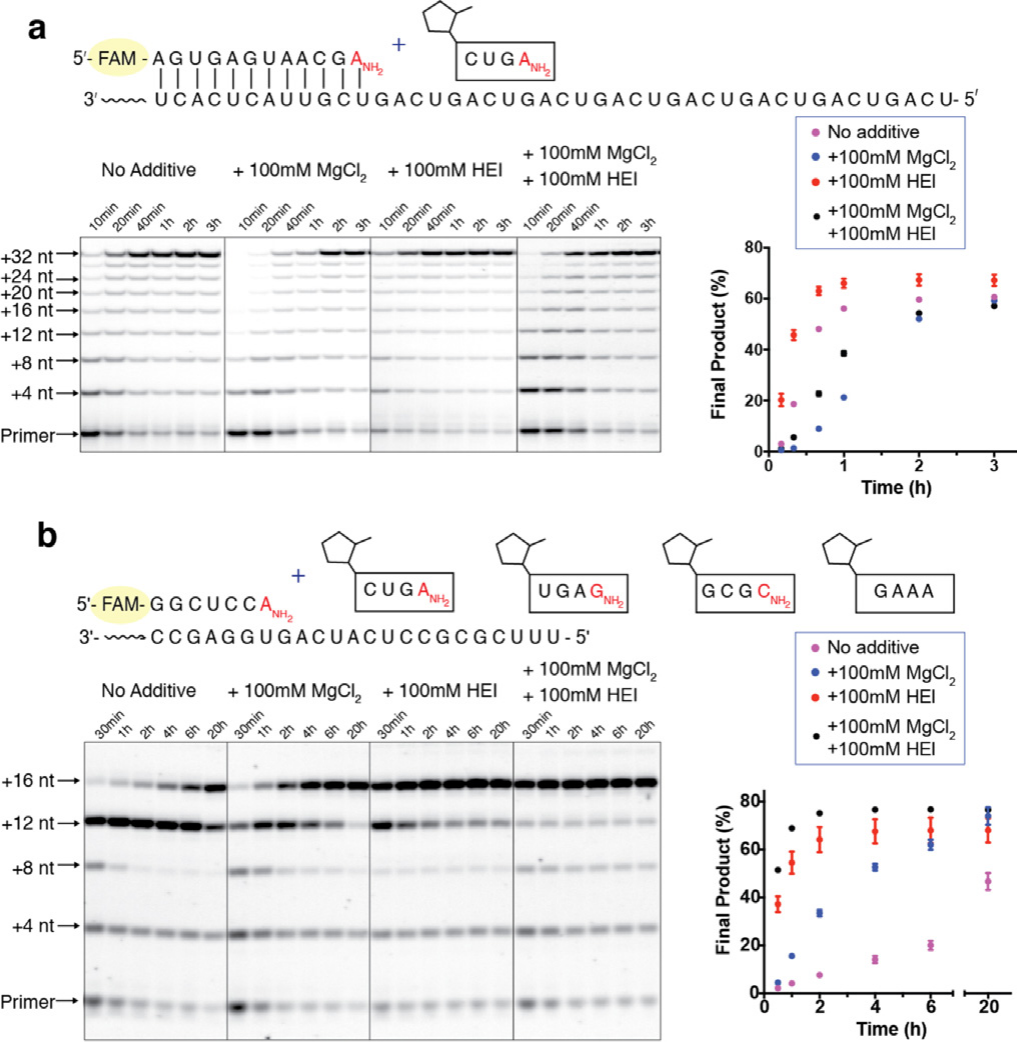
Non‐enzymatic oligonucleotide ligation via N3′‐P5′ linkage formation can rapidly copy a long RNA template. a) Copying of a 32 nt long repeating sequence by the tetramer 2‐MeImp‐CUGA${}_{\mathrm{NH}{}_{2}}$ . Reactions contained 1.5 μm primer, 3 μm template, 200 mm HEPES, pH 8.0, 400 μm 2‐MeImp‐CUGA${}_{\mathrm{NH}{}_{2}}$ . b) Copying of a 16 nt long sequence by ligation of four different tetranucleotides. Reactions contained 2 μm primer, 3 μm template, 200 mm HEPES, pH 8.0, 50 μm 2‐MeImp‐CUGA${}_{\mathrm{NH}{}_{2}}$ , 50 μm 2‐MeImp‐UGAG${}_{\mathrm{NH}{}_{2}}$ , 50 μm 2‐MeImp‐GCGC${}_{\mathrm{NH}{}_{2}}$ , 50 μm 2‐MeImp‐GAAA. Data points are reported as the mean±s.d., *n*≥3.

Encouraged by the greatly enhanced ligation rate resulting from the combination of a 3′‐amino nucleophile and the HEI organocatalyst, we applied these advances to the copying of RNA templates by multistep ligation. We first tested the copying of a 32 nt RNA template containing eight 5′‐UCAG‐3′ repeats (Figure 3 a) in the presence of 400 μm of the tetramer 2‐MeImp‐CUGA‐3′‐NH_2_. With HEI, we observed an approximately 20 % yield of full‐length product within 10 minutes and over 60 % by 40 minutes. Consistent with our previous results, at this saturating concentration of tetramer, Mg^2+^ inhibited ligation, leading to increased levels of a ladder of stalled products corresponding to the stepwise addition of tetramers. After two hours, we did not observe any further increase in the yield of full‐length product, and the stalled products remained unchanged in intensity. To test the hypothesis that the stalled products arise from templates occupied by hydrolyzed (i.e. unactivated) downstream ligator, we repeated the ligation experiments but added multiple aliquots of the coupling reagent EDC after the initial stage of the reaction was largely complete. In the absence of Mg^2+^, this strongly decreased the stalled products and significantly increased the yield of full‐length product (Figure S10). The effect of EDC was much smaller in the presence of Mg^2+^. We suggest that more prebiotically plausible chemistry for the re‐activation of ligator oligonucleotides that have become deactivated due to hydrolysis, such as the recently described isonitrile activation pathway,^32^ might also significantly increase the yield of full‐length product in multistep template‐copying reactions.

To explore the potential of multistep chemical ligation for the copying of arbitrary non‐repeating template sequences, we examined the copying of a 16 nt non‐repeating template in the presence of four different tetramers at 50 μm each (Figure 3 b). As before, the full‐length product was formed rapidly in the presence of HEI, with approximately 60 % yield of full‐length product observed at 2 hours. In this system, Mg^2+^ significantly improved the rate of full‐length product formation. This effect was particularly notable for the addition of the last tetramer 2‐MeImp‐GAAA, presumably due to the Mg^2+^ facilitated binding of this A‐rich ligator. Thus, the combination of a 3′‐amino nucleophile, HEI catalysis, and Mg^2+^ enables the rapid and high yielding copying of mixed‐sequence RNA templates.

**Figure 4:**
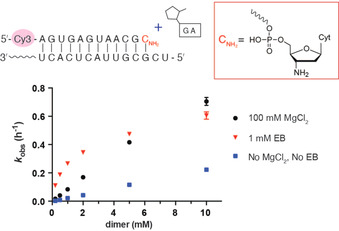
Ethidium bromide enhances the ligation of a 2‐methylimidazole activated dinucleotide to a 3′‐amino primer. Reactions contained 2 μm primer, 4 μm template, 200 mm HEPES, pH 8.0, 0.2–10 mm 2‐MeImp‐GA. Data points are reported as the mean±s.d., *n*≥3.

Although multistep ligation allows the copying of RNA templates, the thermal stability of the resulting long RNA duplexes makes it difficult to separate the strands and then keep them apart long enough for the product strand to act as a template for the next round of copying.^9^ This problem of product inhibition has long been investigated, since it cannot be overcome simply by a denaturation–renaturation approach.^33^ We therefore asked whether multistep ligation could be directed by short RNA template splints that could dissociate more easily. Enzyme‐catalyzed splinted ligation^34^ is widely used to generate RNA products that are much longer than the starting materials, thus hinting at a plausible chemical pathway for the emergence of the first long ribozyme sequences or templates in the pre‐RNA world. The Hud group has demonstrated *N‐*cyano‐imidazole‐driven ligation of DNA tetramers into long polymers with the help of intercalators to stabilize a tiled tetramer array held together by base pairing between two nucleotide overhangs. Unfortunately, this approach did not work with RNA tetramers.^35^ We therefore decided to investigate the potential effect of ethidium bromide (EB) on the assembly of long oligonucleotides through the stabilization of short overlaps of 3′‐amino terminated RNA oligonucleotides. We first examined the ligation of a 2‐methylimidazole‐activated dinucleotide to a 3′‐amino RNA primer (Figure 4). The rate of ligation without any additive is very slow. Even at 10 mm dimer, the rate is only around 0.2 h^−1^, and with the addition of 100 mm Mg^2+^, the rate elevated to around 0.7 h^−1^. Compared with the much higher rates observed with a tetramer ligator (Figure 1 c), we conclude that the weak binding affinity of the dinucleotide is the limiting factor for the ligation reaction. Since 10 mm dimer may be too high a concentration to be prebiotically plausible, the ligation of 10 mm tetramers to each other with two‐base‐pair overlaps may also be unrealistic. We therefore tested the dinucleotide ligation reaction in the presence of 1 mm EB (Figure 4, red triangles). We found that at relatively low concentrations of dinucleotide ligator, EB significantly enhances the ligation rate, by as much as 25‐fold (Figure 4).

**Figure 5:**
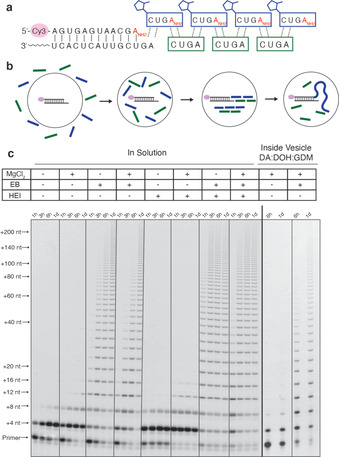
Assembly of long oligonucleotides in solution and inside vesicles by tetramer ligation. a) The splint template is non‐activated tetramer 5′‐AGUC‐3′. The ligator is 5′‐MeImp‐CUGA${}_{\mathrm{NH}{}_{2}}$ . Cy3‐labeled primer with a GA overhang is used to visualize the reaction. b) Scheme of the reaction inside vesicles. c) Representative gels are shown for reactions in solution and inside DA/DOH/GDM vesicles. Reactions in solution contained 4 μm primer, 6 μm template, 0.5 mm AGUC, 0.5 mm CUGA${}_{\mathrm{NH}{}_{2}}$ , 200 mm HEPES, pH 8.0, 0 or 100 mm MgCl_2_, 0 or 1 mm EB, 0 or 100 mm HEI, at room temperature. Reactions in vesicles were performed with 200 mm Bicine, pH 8.5, and the addition of 0.5 mm AGUC, 0.5 mm CUGA${}_{\mathrm{NH}{}_{2}}$ , 50 mm MgCl_2_, 200 mm sodium citrate, 0 or 1 mm EB outside the vesicle.

Encouraged by the effect of EB on dinucleotide ligation, we proceeded to examine the assembly of long oligonucleotides from tetramers, using the unactivated tetramer 5′‐AGUC‐3′ as a splint or template, and the activated tetramer 2‐MeImp‐CUGA‐3′‐NH_2_ as the oligonucleotide to be polymerized (Figure 5 a). To monitor the non‐enzymatic ligation reaction, we used a 5′‐fluorophore‐labeled primer containing a 3′‐amino terminus hybridized to a template with a 5′‐AG overhang. We conducted these experiments both in solution and inside vesicles, which were formed by the mixture of 4:1:1 (v/v/v) of decanoic acid/decanol/monocaprin (DA/DOH/GDM; Figure 5 b). The primer template duplex was encapsulated inside the vesicles, and the vesicles were purified away from unencapsulated primer–template complexes. We then added the tetramer splints, ligators, 50 mm MgCl_2_, and 200 mm sodium citrate to the solution outside the vesicles. We have previously reported that the DA/DOH/GDM vesicles are permeable to tetranucleotides in the presence of 50 mm MgCl_2_ and 200 mm sodium citrate.^36^ We found that both in solution and inside vesicles, ligation products up to 200 nt long formed within 1 day with the assistance of EB. This experiment serves as a proof‐of‐concept that non‐enzymatic ligation is capable of generating long oligomers without pre‐existing long RNA templates.

## Conclusion

In summary, we have demonstrated the efficient copying of RNA templates by non‐enzymatic multistep ligation reactions. We have also demonstrated the assembly of long RNA templates using tetramer RNA splints in solution and inside model protocells. Although 3′‐amino RNA primers and ligators may not be prebiotically plausible, such modified oligonucleotides have been widely used to model the potential behavior of RNA on longer time scales, or under different conditions. Our encouraging results with regard to the assembly and copying of 3′‐amino modified RNA oligonucleotides suggest several areas for future research. For example, we suggest that template‐directed oligomerization of 3′‐amino monomers followed by the ligation of the resulting oligonucleotides may enable the copying of mixed sequence templates in a way that is not dependent on a supply of pre‐existing defined‐sequence oligonucleotide substrates. The studies on ligation presented here, together with our previous studies on the polymerization of 2‐aminoimidazole activated 3′‐amino‐2′,3′‐dideoxy‐ribonucleotide monomers, serve as a framework for future investigations of the copying of RNA templates using substrates with the more nucleophilic 3′‐amino moiety. Until the ligation of canonical RNA oligonucleotides can be significantly improved, 3′‐amino‐modified oligonucleotides provide the best available model system for the study of multistep non‐enzymatic ligation as a path to template copying.

Other avenues to enhance template‐copying chemistry should also be explored. Efforts to identify prebiotically plausible RNA intercalators would be quite useful. We used HEI as an organocatalyst, but prebiotically plausible N‐alkyl and N‐acyl imidazoles should also be examined. The identification of catalysts that could facilitate deprotonation of the 3′‐hydroxyl would of course be highly significant. As an alternative strategy, the search for prebiotically plausible pathways to 3′‐amino modified RNAs should not be neglected. Finally, the assembly of functional ligase ribozymes^37^ by splinted chemical ligation would be an important step in the transition from non‐enzymatic ligation to enzymatic ligation. In the “RNA world”, such ligase ribozymes might contribute critically to efficient pathways for genomic replication. Understanding the transition from non‐enzymatic to ribozyme catalyzed pathways remains a major challenge.

## Conflict of interest

The authors declare no conflict of interest.

## Supporting information

As a service to our authors and readers, this journal provides supporting information supplied by the authors. Such materials are peer reviewed and may be re‐organized for online delivery, but are not copy‐edited or typeset. Technical support issues arising from supporting information (other than missing files) should be addressed to the authors.

Supplementary
