# Supplementary material for: Template‐Directed Copying of RNA by Non‐enzymatic Ligation
Source: Angew Chem Int Ed Engl. 2020 Aug 13;59(36):15682–7. doi: 10.1002/anie.202004934 (PMC7496532; doi:10.1002/anie.202004934)
Supplement: Supplementary file 1 — Supplementary [file ANIE-59-15682-s001.pdf]

Angewandte Chemie
Eine Zeitschrift der Gesellschaft Deutscher Chemiker

Supporting Information

Template-Directed Copying of RNA by Non-enzymatic Ligation**

*Lijun Zhou+, Derek K. O'Flaherty+, and Jack W. Szostak**

anie_202004934_sm_miscellaneous_information.pdf

Author Contributions

L.Z. Conceptualization: Equal; Data curation: Equal; Formal analysis: Equal; Visualization: Equal; Writing—Original Draft: Equal; Writing—Review & Editing: Equal
D.O. Conceptualization: Equal; Data curation: Equal; Formal analysis: Equal; Visualization: Equal; Writing—Original Draft: Equal; Writing—Review & Editing: Equal
J.S. Conceptualization: Supporting; Investigation: Lead; Resources: Lead; Supervision: Lead; Writing—Original Draft: Supporting; Writing—Review & Editing: Supporting.

Table of Contents

Experimental Procedures........ 3
Supplementary Figures 1-10........ 4
References........ 14
Author Contributions........ 14

Experimental Procedures

Chemical synthesis of phosphoramidites

Protected 3′-amino phosphoramidites were prepared according to previously reported procedures[1]. 3′-Amino-2′,3′-dideoxycytidine was purchased from Carbosynth (Campton, UK). 3′-Amino-2′,3′-dideoxyguanosine, 3′-amino-2′,3′-dideoxyadenosine and 3′-amino-2′,3′-dideoxythymidine were purchased from Fisher/Alfa Aesar (Haverhill, MA). Other chemical reagents were purchased from Sigma-Aldrich.

Solid phase synthesis of oligonucleotides

Oligonucleotides were prepared by solid-phase synthesis on an Expedite 8909 or MerMade 6 DNA/RNA synthesizer (Bioautomation, Plano, TX). Chemical reagents were purchased from Glen Research (Sterling, VA) and Chemgenes (Wilmington, MA). RNAs were deprotected by standard methods. Non-dye labeled oligonucleotides were purified by GlenPak columns. Dye-labeled oligos were purified by polyacrylamide gel electrophoresis and desalted by Sep-Pak C18 cartridge from Waters (Milford, MA).

Activated Oligonucleotides

5′-phosphorylated oligonucleotides were activated with 2-methylimidazole as previously described[2,3]. Activated tetranucleotides were purified on Agilent ZORBAX PrepHT columns (Eclipse XDB-C18, 250 × 21.2 mm, 7 µm particle size, P.N. 977250-102), at a flow rate of 15 ml/min, using gradient elution between (A) aqueous 20 mM triethylammonium bicarbonate, pH 8.0, and (B) acetonitrile. Activated tetramers were separated from unactivated tetramers with a gradient of 6% to 11 % B over 14 minutes. Activated decanucleotides were purified on Agilent ZORBAX analytical columns (Eclipse Eclipse Plus C18, 250 × 4.6mm, 5 µm particle size, P.N. 959990-902), at a flow rate of 1 ml/min, using gradient elution between buffers A and B as above. Activated decamers were separated from unactivated decamers with a gradient of 6% to 10 % B over 17 minutes.

High-resolution mass spectrometry

Oligonucleotides were analyzed on an Agilent 1200 HPLC coupled to an Agilent 6230 TOF mass spectrometer. Samples were resolved by IP-RP-HPLC on a 100 mm × 1 mm Xbridge C18 column with 3.5 µm particle size (Waters, Milford, MA) using gradient elution between (A) aqueous 200 mM 1,1,1,3,3,3-hexafluoro-2- propanol with 1.25 mM triethylamine, pH 7.0, and (B) methanol. Samples were analyzed in negative mode from 239 m/z to 3200 m/z with a scan rate of 1 spectrum/s.

Non-enzymatic Ligation Reaction

Unless otherwise indicated, non-enzymatic primer extensions were performed according to previously reported[4] procedures except that we used ligator oligonucleotides instead of mononucleotides. At each time point, an 0.5 µl aliquot of the reaction mixture was added to 25 µl of quenching buffer, containing 10 mM EDTA, 5-10 µM complementary strand RNA, 0.5 x TBE buffer and 90 % (v/v) formamide. Quenched samples were heated to 95 °C for 1 min and cooled quickly to room temperature (typically 25 °C). Primer extension products were resolved by 20 % (19:1) denaturing PAGE with 7 M urea. For $MgCl_2$ titration experiments, the quenching buffer contained 50 mM EDTA instead of 10 mM. For the experiments in Fig. S6 and Fig. S7, the quenching buffer was pre-heated to 100 °C, to ensure immediate quenching, because at room temperature, the N3'–P5' ligation of the decamer ligator continues in formamide or 8M urea.

Non-enzymatic Ligation Reaction inside vesicles

The process of vesicle preparation has been previously reported[5]. 2.4 µl Decanoic Acid (Nu-Chek #N-10-A), 0.6 µl decanol (Nu-Chek # A-610) and 0.75 µl Monocaprin (Nu-Chek # M-124) were mixed at 65 °C. Then 125 µl pre-annealed primer (13 µM) / template (22 µM) in 200 mM Na^+ bicine, pH 8.5, 160 µM calcein was added to the mixed lipid. The mixture was mixed rigorously by vortexing for 1 minute, and then tumbled for 12 h. The vesicles were extruded to 200 nm and again tumbled overnight. The unencapsulated primer/template were removed by a Sepharose 4B size exclusion column.

Ligation reactionS were initiated by adding 0.5 mM tetramer splint and 0.5 mM activated tetramer to the purified vesicleS, followed by pre-mixed Mg:Citrate (final concentration 50mM:200mM), and 1 mM EB. Then tumbling of the reaction mixture was continued.

At each time point, 20 µl of the reaction mixture was removed, and the vesicle fraction was purified away from any leaked oligonucleotides on a Sepharose 4B size exclusion column. The purified vesicles were collected, lysed and the nucleic acids precipitated in 750 µl water, 20 µl 5 mg/ml glycogen, 30 µl 20% (V/V) triton X-100 and 4 ml ethanol. After incubation at -20 °C for 2 hrs, the sample was centrifuged at 3700 rpm for 45 mins. The supernatant was discarded, and the pellet washed with 80% ethanol in water. The pellet was air-dried, resuspended in gel loading buffer (8M urea, 1x TBE, 20 mM EDTA) and then analyzed by 10% (19:1) denaturing PAGE with 7M urea.

Supplementary Figures

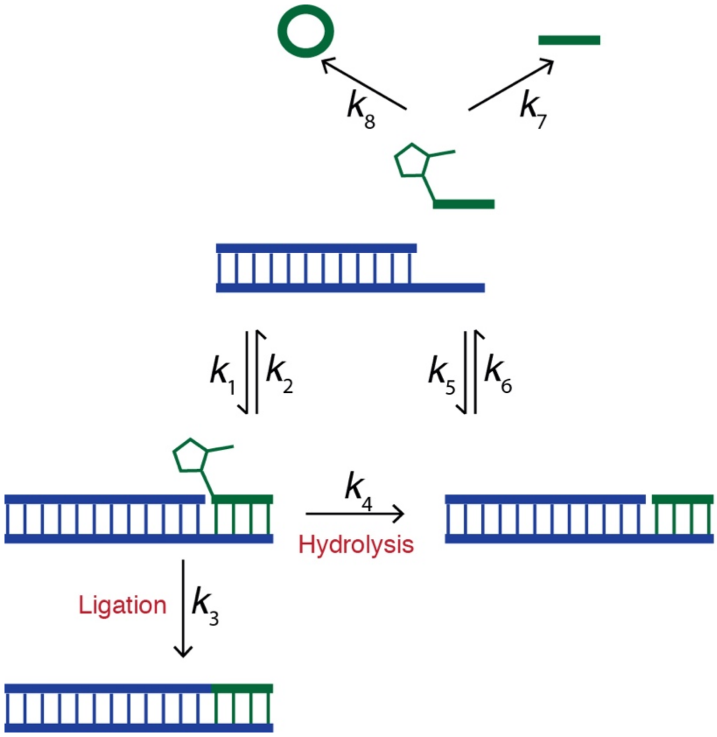

Figure S1: Kinetic scheme for reactions involved in non-enzymatic oligonucleotide ligation. Hydrolysis reactions, both on and off template, compete with the ligation reaction. Reversible binding of the ligators to the template is followed by competition between ligation and hydrolysis. The fraction of the primer/template complex that ultimately becomes ligated depends upon the dissociation of inactive (hydrolyzed) ligators to allow new incoming activated ligators to bind the template.

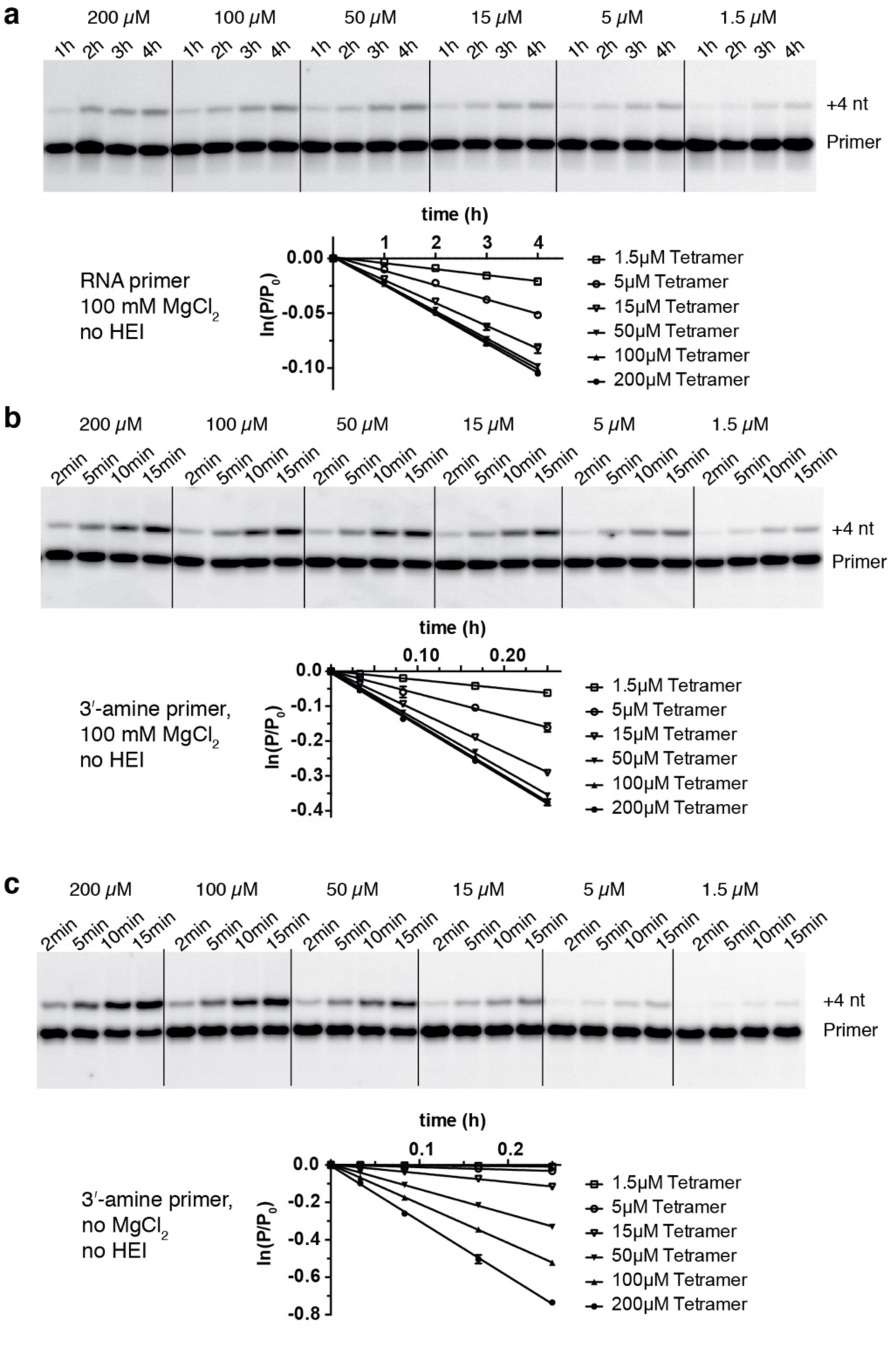

Figure S2: Representative PAGE data and plots of $\ln(P/P_0)$ vs. time for the ligation reaction kinetics described in Figures 1b and1c. (a) Ligation of the all RNA primer to the activated tetramer at the indicated concentrations, in the presence of 100 mM $MgCl_2$, in the absence of HEI. (b) Ligation of the 3′-amino terminated primer to the activated tetramer at the indicated concentrations, in the presence of 100 mM $MgCl_2$, in the absence of HEI. (c) Ligation of the 3′-amino terminated primer to the activated tetramer at the indicated concentrations, in the absence of both $MgCl_2$ and HEI.

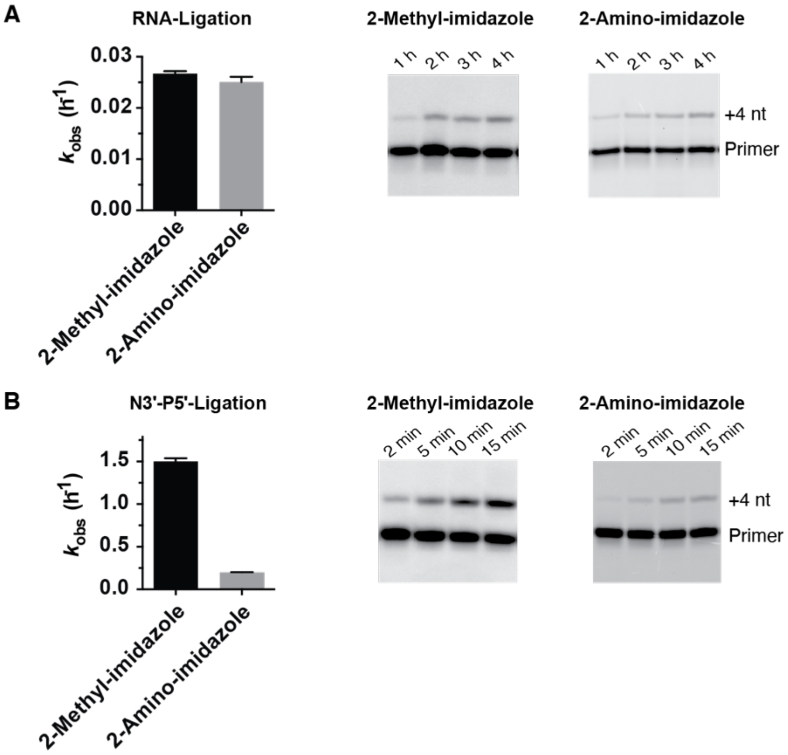

Figure S3: Ligation rates of a tetramer activated with two different leaving groups. All experiments were carried out with 2 μM primer, 4 μM template, 200 mM HEPES, pH 8.0, 100 mM $MgCl_2$ and either 200 μM 2-MI-CUGA or 2-AI-CUGA as indicated. (A) 3′-OH primer. (B) 3′-NH_2 primer. The ligation rates of both substrates are quite similar with a 3′-OH primer, but ligation of the 2AI-activated tetramer was 7-fold slower than the 2MI activated tetramer. We attribute these results to the fact that 2-amino-imidazole is a poorer leaving group than 2-methyl-imidazole. In the case of the 3′-OH primer, the ligation rates are very slow and the rate limiting step in the reaction is the deprotonation of the 3′-OH. However, once the 3′-OH is deprotonated the alkoxide is such a strong nucleophile that the identity of the leaving group makes little difference. In contrast, the 3′-NH_2 is a good nucleophile but not nearly as strong as an alkoxide, so the stability of the leaving group has a significant influence on the reaction rate.

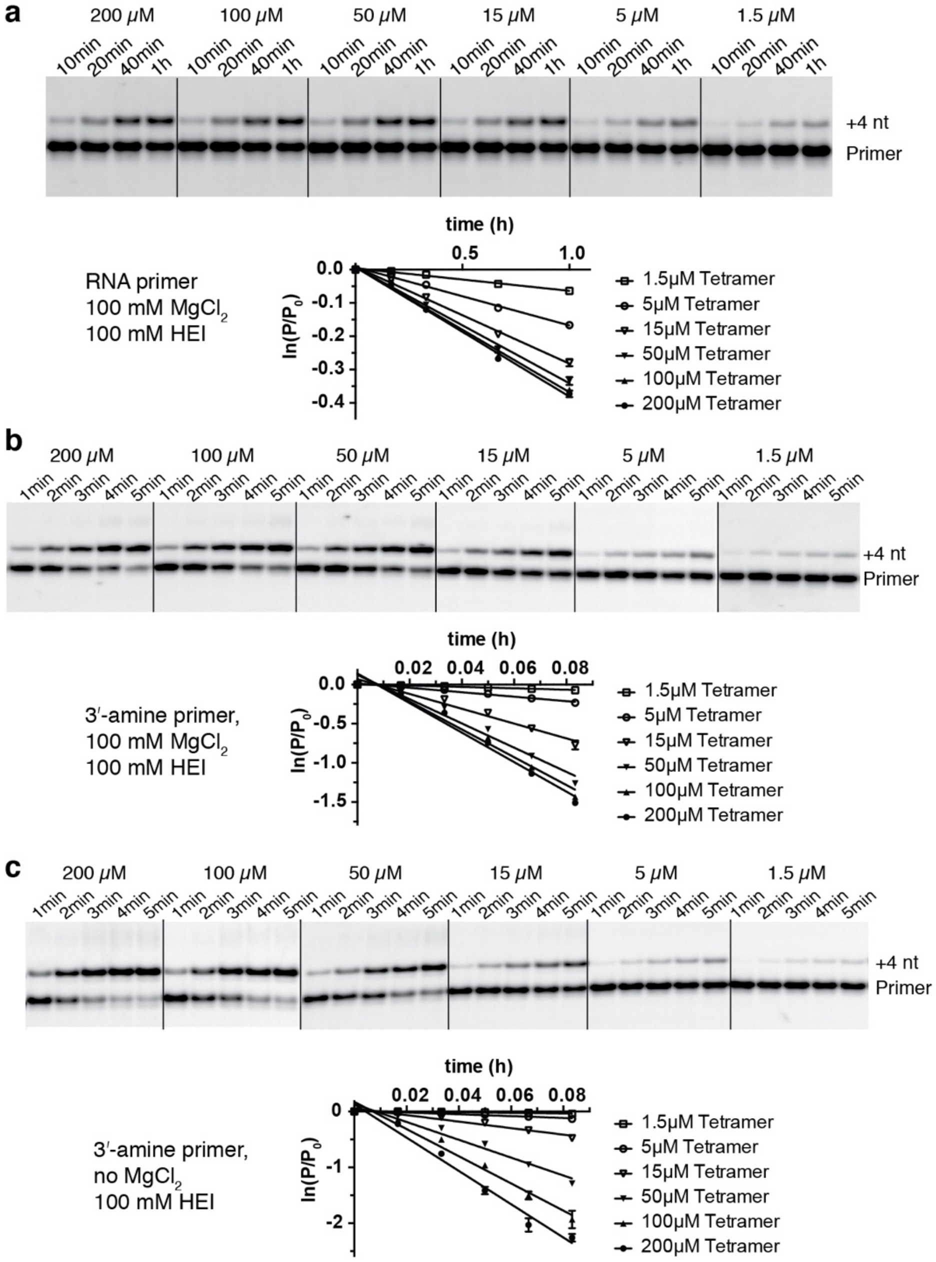

Figure S4: Representative PAGE data and plots of ln(P/P0) vs. time for the ligation reaction kinetics described in Figures 1d and 1e. (a) Ligation of the all RNA primer to the activated tetramer at the indicated concentrations, in the presence of 100 mM $MgCl_2$ and 100 mM HEI. (b) Ligation of the 3′-amino terminated primer to the activated tetramer at the indicated concentrations, in the presence of 100 mM $MgCl_2$ and 100 mM HEI. (c) Ligation of the 3′-amino terminated primer to the activated tetramer at the indicated concentrations, with 0 mM $MgCl_2$ and 100 mM HEI.

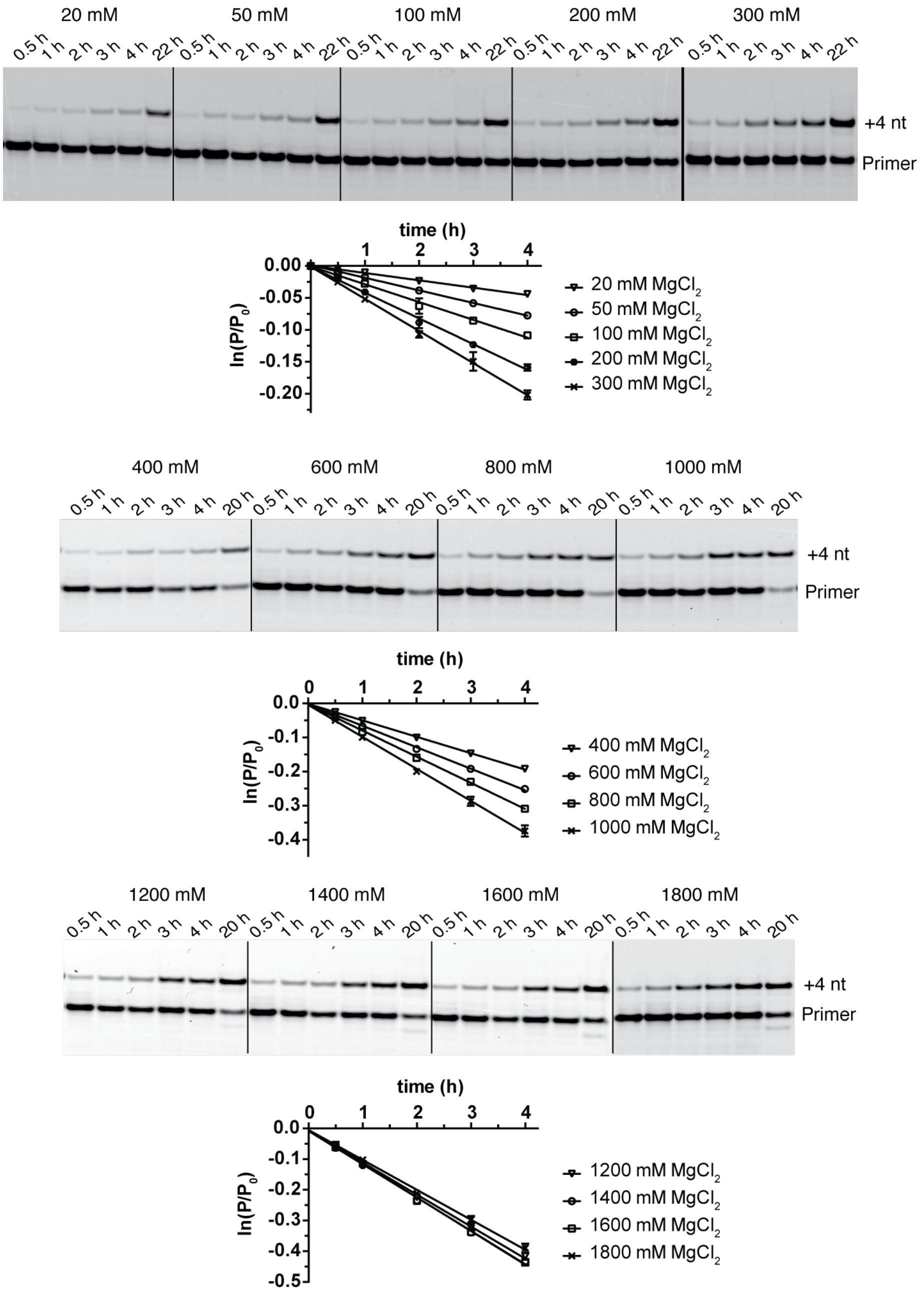

Figure S5: Representative PAGE data and plots of ln(P/P_0) vs. time for the ligation reaction kinetics described in Figure 2a.

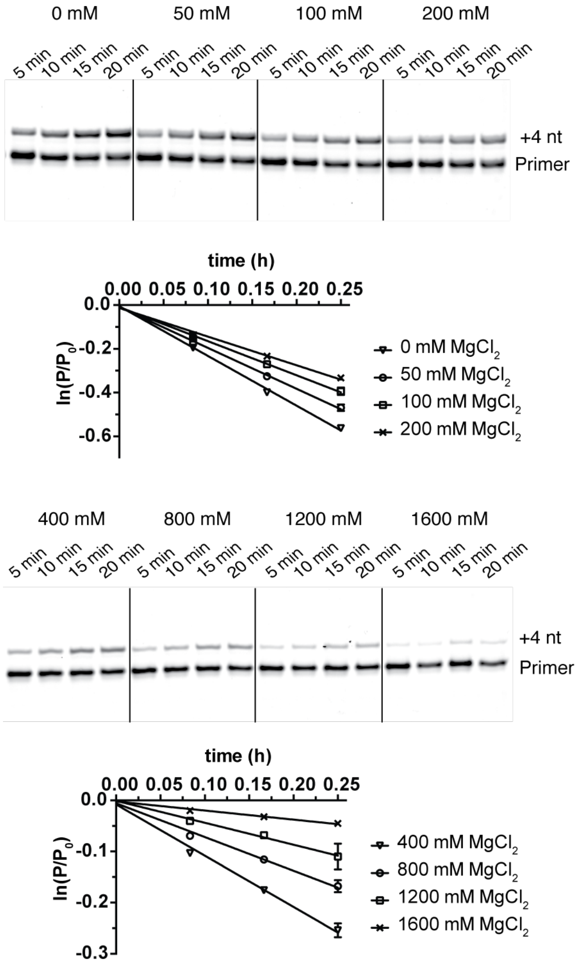

Figure S6: Representative PAGE data and plots of $\ln(P/P_0)$ vs. time for the ligation reaction kinetics described in Figure 2b.

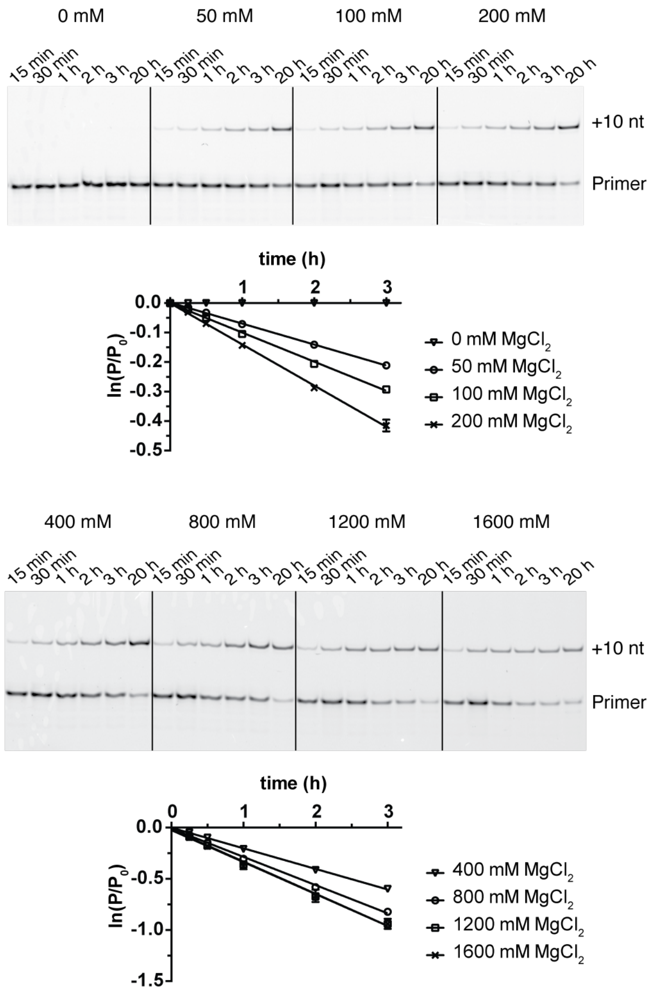

Figure S7: Representative PAGE data and plots of ln(P/P_0) vs. time for the ligation reaction kinetics described in Figure 2c.

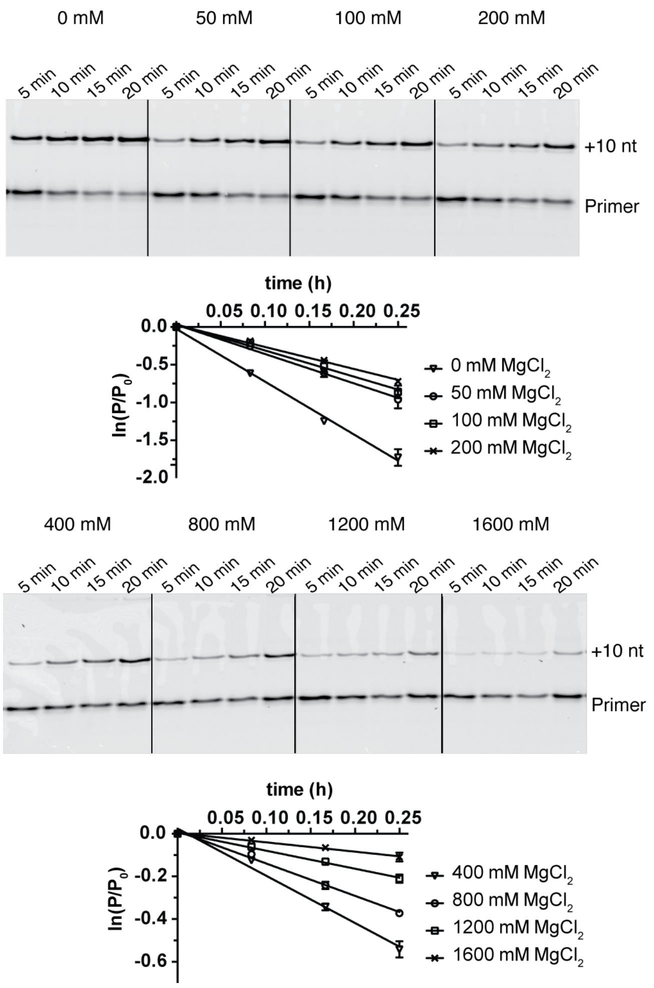

Figure S8: Representative PAGE data and plots of ln(P/P_0) vs. time for the ligation reaction kinetics described in Figure 2d.

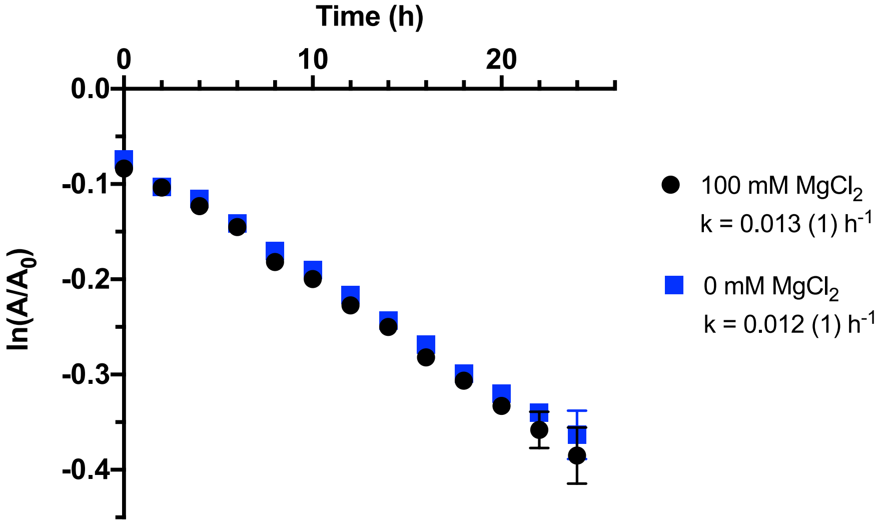

Figure S9: Hydrolysis of 2-methyl-imidazole activated ligator tetramer in the presence and absence of Mg^{2+}. 0.5 mM 2-MeImp-CUGA in 200 mM HEPES, pH 8.0 was incubated with 100 mM $MgCl_2$ (black circle) or 0 mM $MgCl_2$ (blue square). The samples were analyzed by analytical HPLC C-18 column. The peaks were integrated, and the hydrolysis rates were calculated as pseudo-first order reactions.

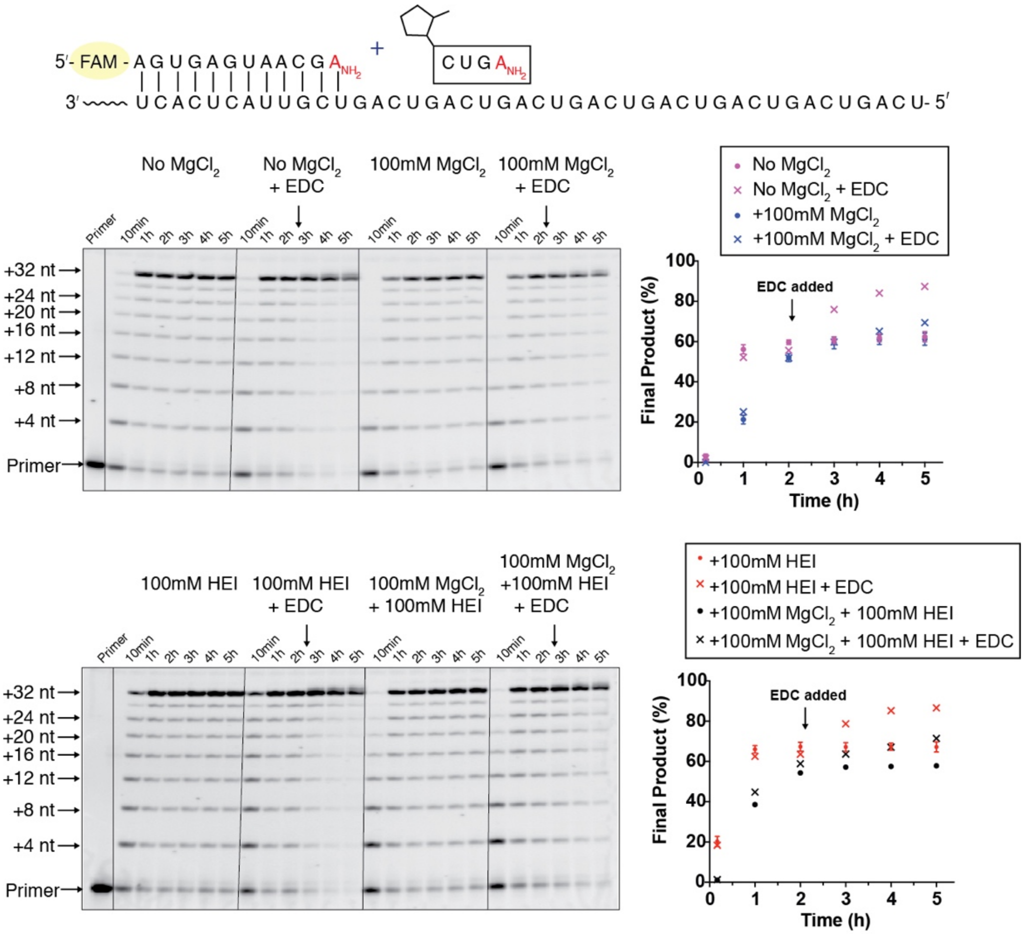

Figure S10: Ligation reactions as in Figure 3a except with the addition of 150 mM EDC after the 2h, 3h and 4h time points.

References

[1] V. S. Lelyveld, D. K. O'Flaherty, L. Zhou, E. C. Izgu, J. W. Szostak, *Nucleic Acids Res.* **2019**, *47*, 8941–8949.

[2] N. Prywes, J. C. Blain, F. Del Frate, J. W. Szostak, *Elife* **2016**, *5*, 1–14.

[3] G. F. Joyce, T. Inoue, L. E. Orgel, *J. Mol. Biol.* **1984**, *176*, 279–306.

[4] L. Li, N. Prywes, C. P. Tam, D. K. Oflaherty, V. S. Lelyveld, E. C. Izgu, A. Pal, J. W. Szostak, *J. Am. Chem. Soc.* **2017**, *139*, 1810–1813.

[5] D. K. O'Flaherty, N. P. Kamat, F. N. Mirza, L. Li, N. Prywes, J. W. Szostak, *J. Am. Chem. Soc.* **2018**, *140*, 5171–5178.

Author Contributions

L.Z., D. K. O. and J.W.S. planned the experiments. L.Z. and D.K.O. synthesized oligonucleotides, carried out the experiments and analyzed the data with equal contribution. J. W. S. obtained the funding and supervised the project. L.Z., D.K.O. and J.W.S wrote the manuscript.
